# Quantitative Measurements in the Human Hippocampus and Related Areas: Correspondence between Ex-Vivo MRI and Histological Preparations

**DOI:** 10.1371/journal.pone.0130314

**Published:** 2015-06-22

**Authors:** José Carlos Delgado-González, Francisco Mansilla-Legorburo, José Florensa-Vila, Ana María Insausti, Antonio Viñuela, Teresa Tuñón-Alvarez, Marcos Cruz, Alicia Mohedano-Moriano, Ricardo Insausti, Emilio Artacho-Pérula

**Affiliations:** 1 Human Neuroanatomy Laboratory and C.R.I.B., School of Medicine, University of Castilla-La Mancha, Albacete, Spain; 2 Radiology Service, Magnetic Resonance Unit, Complejo Hospitalario Universitario de Albacete (CHUA), Albacete, Spain; 3 Radiodiagnostic Service, Hospital Nacional de Parapléjicos (HNP), Toledo, Spain; 4 Department of Health, Physical Therapy School, Public University of Navarra, Tudela, Spain; 5 School of Advanced Education, Research and Accreditation, Castellón de la Plana, Spain; 6 Pathology Service, Hospital Complex of Navarre, Pamplona, Spain; 7 Department of Mathematics, Statistics and Computation, University of Cantabria, Santander, Spain; University of Modena and Reggio Emilia, ITALY

## Abstract

The decrease of volume estimates in different structures of the medial temporal lobe related to memory correlate with the decline of cognitive functions in neurodegenerative diseases. This study presents data on the association between MRI quantitative parameters of medial temporal lobe structures and their quantitative estimate in microscopic examination. Twelve control cases had ex-vivo MRI, and thereafter, the temporal lobe of both hemispheres was sectioned from the pole as far as the level of the splenium of the corpus callosum. Nissl stain was used to establish anatomical boundaries between structures in the medial temporal lobe. The study included morphometrical and stereological estimates of the amygdaloid complex, hippocampus, and temporal horn of the lateral ventricle, as well as different regions of grey and white matter in the temporal lobe. Data showed a close association between morphometric MRI images values and those based on the histological determination of boundaries. Only values in perimeter and circularity of the piamater were different. This correspondence is also revealed by the stereological study, although irregular compartments resulted in a lesser agreement. Neither age (< 65 yr and > 65yr) nor hemisphere had any effect. Our results indicate that ex-vivo MRI is highly associated with quantitative information gathered by histological examination, and these data could be used as structural MRI biomarker in neurodegenerative diseases.

## Introduction

The medial temporal lobe (MTL) is considered to comprise the Hippocampal Formation (HF) and the parahippocampal region (PHR). The HF is subdivided into Dentate Gyrus, CA1, CA2 and CA3 fields, Subiculum, Presubiculum, Parasubiculum and Entorhinal cortex. The PHR is composed of the temporopolar, perirhinal and parahippocampal cortices, which lie adjacent to the HF and surround it as a cortical strip. The MTL is crucial in the network supporting encoding and consolidation of declarative memory [1]. MTL lesions in humans produces memory deficits; moreover, experimental studies in animals demonstrate the pivotal role of limbic medial temporal structures in memory [2]. The PHR is activated during recognition judgments [3, 4], and is considered as the anatomical substrate of relational memory by linking individual stimuli to context [5].

The MTL in general shows atrophy from the eight decade on [6], and in particular, the HF is affected. For instance, while the entorhinal cortex (EC) decreases 5% in volume, in subjects under 70 years, it reaches 20% over 70 years [7]. Furthermore, the number of neurons in the HF [8] is an important indicator to assess brain changes related to memory functions in aging and neurodegenerative diseases such as Alzheimer’s disease (AD). This neurodegenerative disease affects differentially regions of the MTL in a distinct pattern: while rostromedial EC shows a scarce neuronal loss, the caudal portion of the EC presents an important reduction in neuron number, as well as in perirhinal cortex, subiculum and dentate gyrus [9, 10]. In addition to the neurodegeneration pattern, global changes, as well as hemispheric differences have been also reported in other neurological diseases such as temporal lobe epilepsy, postraumatic stress disorder, depressive disorders, Down syndrome, or autism [11–13]. More concerted comparison with pathological findings in particular in AD [6, 14] can be achieved through the comparison of morphological parameters between ex-vivo MRI images and histological sections of the same cases, also helpful to estimate quantitative features of the MTL and their variability. The effect of age and sex and intra-subject hemispheric asymmetry in different structures of the MTL are also evaluated. The normative values would provide a useful tool for quantitative assessment of structural MRI, in order to help in the diagnosis of neurological diseases in which a variation in MTL volume is produced.

The present study aims at finding both morphometrical and stereological estimates of the MTL as well as the remainder of temporal cortex and amygdaloid complex in a series of control cases, both at ex-vivo MRI and histological examination as a means of validation of in-vivo MRI examination. Thereby, a simple procedure would facilitate a clear, unbiased, and efficient method for neuroradiological examinations in the MTL structures.

## Materials and Methods

### Materials

The study consisted of twelve human brains (five men and seven women, age range, 40 to 90 years) obtained from routine autopsies performed between the years 2001–2002 at the Pathology Department Hospital Complex of Navarre (Spain), according to the local Ethical Committee (Table 1). Informed consent to perform autopsy and study of organic tissues for research use was obtained verbally by the pathologist from the next of kin. Prior to 2004 no written informed consent was required for routine autopsies, but verbal consent was obtained from the next of kin by the pathologist who performed the autopsies, based on the lack of regulation of written informed consent and the research purposes of the project. After the year 2004, written informed consent is mandatory and archived at the Hospital or Brain Bank. Furthermore, the specific research project was approved by the Clinical Investigation Ethical Committee (CEIC), available at the website http://www.chospab.es/investigacion/ceic/intro.htm. Study requirements (with an in-depth analysis of the objectives of the study, proofs and preparation, benefits and risks, confidentiality and data protection, and others) comply with the Helsinki Declaration. The Ethical Committee approved the study as all cases were prior to the year 2004, and the use of biological organs or tissue for scientific research is ethically legitimated, as it is the case in the present study. The Ethical Committee approved this research project.

**Table 1:** Study cases.

Case	Age (years)	Age’s group	Sex	Cause of death
05/01	47	LT	M	Chronic myeloid leukemia; meningitis
08/02	59	LT	M	Acute hepatic encephalopathy by chronic alcoholism*
10/02	57	LT	M	Sudden death; chronic alcoholism*
22/02	61	LT	W	Sudden death; arterial hypertension
35/02	58	LT	W	Celiac disease with diffuse intestinal infiltration
51/01	40	LT	W	Diabetes mellitus
01/02	71	GT	W	Abdominal tumour
11/02	90	GT	M	Lymphoma
32/02	78	GT	W	Sepsis
36/01	83	GT	W	Lymphoma
38/02	83	GT	W	Colon cancer
59/02	75	GT	M	Leukemia

Case indicates the anonymous ordinal number of autopsy. M, man; W, woman. Age’s group: LT (lower than), less or equal to 65 years; GT (greater than), 65 years.

* (asterisk): the two cases marked (with alcoholism) did not differ in results with respect to other cases, without significant changes in results.

The cases were grouped according to the age as LT (lower than), less or equal to 65 years, and GT (greater than), 65 years, based on the study of Insausti *et al*. [6]. The cause of death of the cases was not related to neurological or psychiatric disorders that may have affected the parameters under study.

**Fig 1:**
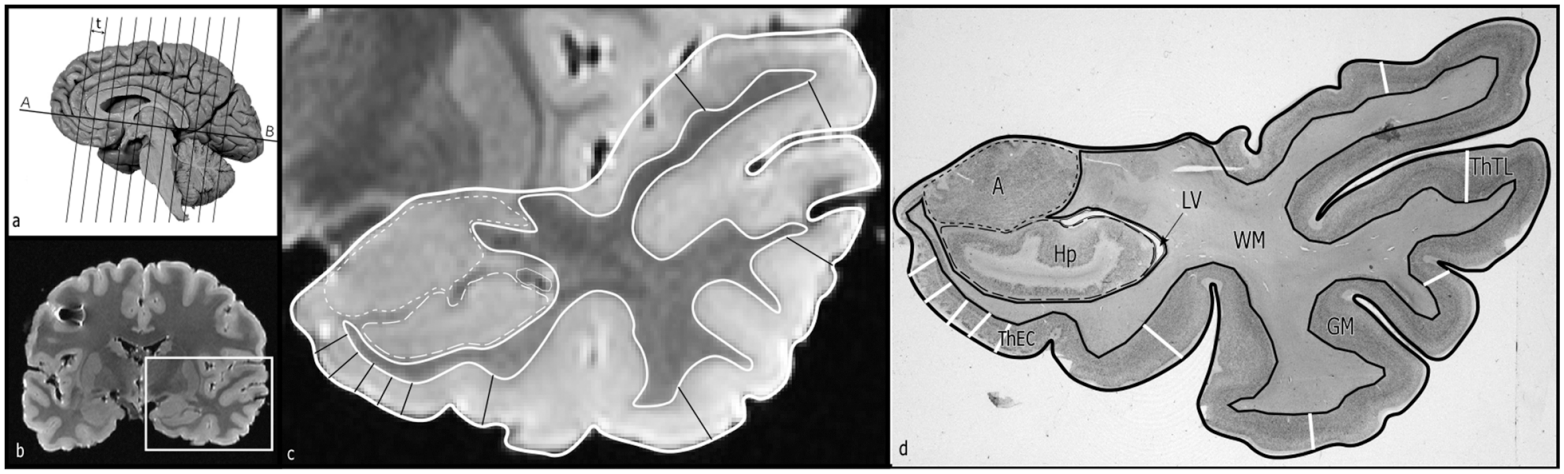
(a) Representation of an hemisphere showing the intercommissural line (A-B line) and the perpendicular sections to obtain consecutive slabs with a thickness of *t*. (b) A MRI at the level of the amygdala, caudal to the limen insulae. (c) MRI depicting the outline the temporal lobe structures. (d) Histological delineation of structures at same level as in panel c. WM: White matter; GM: Grey matter; A: Amygdala; Hp: Hippocampus; LV: Temporal horn of the lateral ventricle; ThEC: scheme with the five measurements of the entorhinal cortex; ThTL: similar representation of the five measurements in the temporal cortex.

The brains were cut in several slabs of approximately 1 cm-thick in the coronal plane, orthogonal to the intercommissural line (according to the standard procedure of [15] (Fig 1); samples were obtained for neuropahological examination. Brain slabs were fixed by immersion in 10% buffered formalin, and subsequently in 4% paraformaldehyde during at least eight weeks. Thereafter, the brain slabs were hold together in a gauze for MRI study using 1.5T magnetic resonance scanner (Gyroscan Intera, Phillips); a gradient SE coronal T1 high resolution sequence was used once brains were placed on the head matrix coil; consecutive MRI sections of the brain were carried out with a thickness of 1.2 mm and an acquisition matrix of 512x512; the effective pixel size was of 0.29 mm.

After MRI study, each temporal lobe was dissected and immersed in a cryoprotectant solution of 10% glycerol with 2% dimethylsulfoxide in phosphate buffer (pH 7.2) for 3 days, and 20% glycerol for 5 additional days. Each slab was serially sectioned in the coronal plane at a thickness of 50 μm in a sliding microtome coupled to a freezing unit. Every 10^th^ section was mounted on gelatin-coated slides and stained with thionin for Nissl histological examination of the different MTL structures analyzed.

### Quantitative study

The quantitative study of the MTL structures was performed in both MRI and histological series of sections. The morphometric and stereological study was carried out with tools of the free software ImageJ which allows the overlap of test systems. MTL structures were identified, first in histological sections and subsequently in MRI series of images. Afterwards, the delimitation of the different structures in the MTL was prepared in series of images arranged in a rostral to caudal sequence, from the temporal pole to the caudal extreme of the hippocampus. The piamater was taken as the outer limit of the temporal cortex. The inner limit was the line between the grey and white matter in the HF and PHR [16].

The amygdaloid complex limits were taken from the level of the limen insulae (rostral limit) to the hippocampal—amygdaloid transitional area (HATA), as the most caudal part of the amygdaloid complex [17]. The subiculum, which forms the anterior limit of the hippocampus in the coronal plane, was recognized as an ovoid mass at the rostral tip of the lateral ventricle, under the amygdaloid complex. The caudal limit of the hippocampus was set at the posterior limit of the posterior parahippocampal cortex, as a mass of ovoid grey matter, at the level of the fimbria, tangentially cut at this level, and in continuation with the posterior crus of the fornix, according to our previous published work [18]. Finally, the measurement of the lateral ventricle started at the onset of the temporal horn of the lateral ventricle, where the amygdaloid complex is ahead to the beginning of the subiculum. The temporal horn measurement ended at the level of the atrium (confluence of the frontal, temporal and occipital horns of the lateral ventricle).

The morphometric parameters obtained in our study were area, perimeter, maximal and minimal Feret’s diameters, and shape descriptors: (1) FFPE (circularity form factor, obtained from: 4 • π • area / perimeter^2^), where values of 1 represented a perfect circle, and lower than 1 for an ellipse and irregular structures, (2) FFEll (elliptic form factor-aspect ratio-, obtained from major axis / minor axis) with values of 1 for a circle and more than 1 for elliptical structures; and (3) FFAR (harmonic form factor—roundness-, obtained by 4 • area / (π • (major axis)^2^)) where a value of 1 represents a circle and an ellipse, and lower than 1 represents irregular structures. These estimators were used in the assessment of (1) the outline of the temporal lobe, (2) white matter outline, (3) amygdaloid complex, (4) HF, and (5) temporal horn of the lateral ventricle. Furthermore, the cortical thickness in each of the gyri of the temporal lobe as far as the PRC, that makes the EC boundary, was assessed in five systematic measurements (Fig 1); the EC was measured from 2 mm behind the limen insulae as far as the EC caudal limit, approximately 1.5 mm behind the last slice containing the gyrus intralimbicus (end of the uncus), just rostral to the lateral geniculate nucleus [16]; the sulcus semiannularis and the collateral sulcus respectively were the landmarks of the medial and lateral boundaries of the EC.

**Fig 2:**
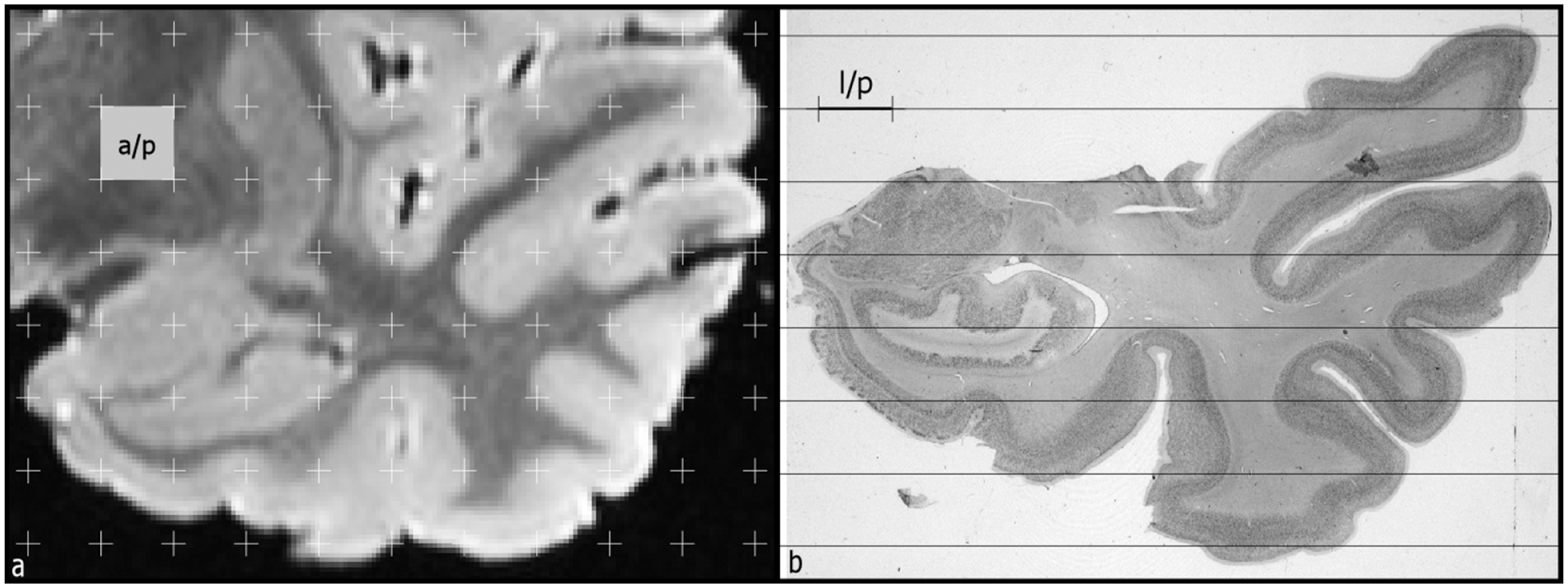
(a) MRI of the temporal lobe. A test system of regularly spaced points is superimposed for the estimation of volume of different structures. The number of points that hit the structure result in the volume, according to the formulae presented in Methods. The area associated to test point is showed (a/p). (b) Histological section stained with thionin at a roughly the same level as in a MRI image, where horizontal lines were superimposed to estimate the intercepts between test lines and each structure of interest (outline of the white matter, grey matter-surface area of the temporal lobe-, profiles of the amygdala, hippocampus and lateral ventricle). The length of test line associated with a test point is also represented.

The stereological study included the estimation of the surface area and volume of the structures analyzed (Fig 2). The volume of both the grey and white matter, amygdaloid complex, HF, as well as the temporal horn of the lateral ventricle was estimated using Cavalieri’s principle [19]. This method allows an efficient and precise volume estimation of a structure regardless of its size and shape, through a series of parallel planes spaced at a constant distance “t”. The final estimation of the volume is obtained according to the following equation:

$$est\left(V\right)\;=\;t•(A_{1}+\;A_{2}+\;A_{3}+\ldots A_{n})$$

in which, *A*_*1*_, *A*_*2*_, *… A*_*n*_ denote the outline sectional area of each structure, and *t*, is the section interval for the *n* consecutive sections. Also, we superimposed an overlay made up of test points regularly spaced for the volume estimation; thus, the sum of points *ΣP* hitting the structure of interest, multiplied by the section interval, *t*, and the area associated with each test point *a/p*, is a useful estimation of the volume. This estimation is expressed as:

est(V) = t•(a/p)•ΣP

The study included an analysis of the accuracy of volume estimates using Cavalieri’s principle and the counting point method according to Cruz-Orive [20]. We also estimated the counting point contribution to the overall estimation of the coefficient of error (CE (*est(V)*)) that is, in general, negligible relative to the contribution of areal measurement between sections.

In addition, the study includes the surface area estimation according to the following equations

$$est\left(S_{V}\right)\;=\;2•(\;1\;/\;\left(l/p\right)\;)•\Sigma I\;/\Sigma P)\;\left(cm^{-1}\right),\;and\;est\left(S\right)\;=\;V•S_{V}\;\left(cm^{2}\right),$$

in which, *l/p* is the length of test line per grid point, *ΣI* is the sum of intersections between the test lines and the profile of the structure and *ΣP* the sum of points hitting the structure. We found limitations in this estimation since test lines must have isotropic orientation and random position in the 3D space.

Once volume and surface area were estimated, the cortical thickness in the 3D space was estimated according to the Eq (67) from Cruz-Orive *et al*. [21], as the ratio of grey matter volume and the average between grey matter and white matter surface areas.

### Statistical study

The quantitative data were statistically analyzed using SPSS/PC+ Statistical Software Package 19.0 (SPSS, Inc., Chicago, Illinois, U.S.A.). The mean value, SD, SEM, and coefficient of variation of each item were computed. The study included a comparison of the mean values for right and left hemispheres, as well as for MRI and histological examination. For this reason, a normal distribution of data was evaluated using the Kolmogorov-Smirnov test. Moreover, a paired t-test was employed to determine the significance between hemispheres and between both set of data, MRI and histological data. Differences related to age’s group and sex using independent t-tests was also examined. A Pearson correlation study was used to correlate quantitative estimators. Also, a multivariate Anova (MANOVA) was carried out with R^®^, independent for volume and surface area parameters. The observations made on each case were conceived as a random vector with five components, corresponding to white and grey matter of temporal cortex, amygdala, hippocampus and temporal horn of the lateral ventricle, respectively. These parameters are robust and meaningful because they are defined in 3D and estimated by stereology. Although the measurements in each case were made separately on every hemisphere, the corresponding data pairs were added up in order to get total brain quantities, mainly because a high correlation among the two hemisphere variables would render them redundant and would thereby impair the MANOVA analysis. Three non-random factors, with two levels each were considered, namely, the method (MRI and physical sections), sex, and age. Separate univariate ANOVA analyses were performed for the study of the cortical thickness.

## Results

**Fig 3:**
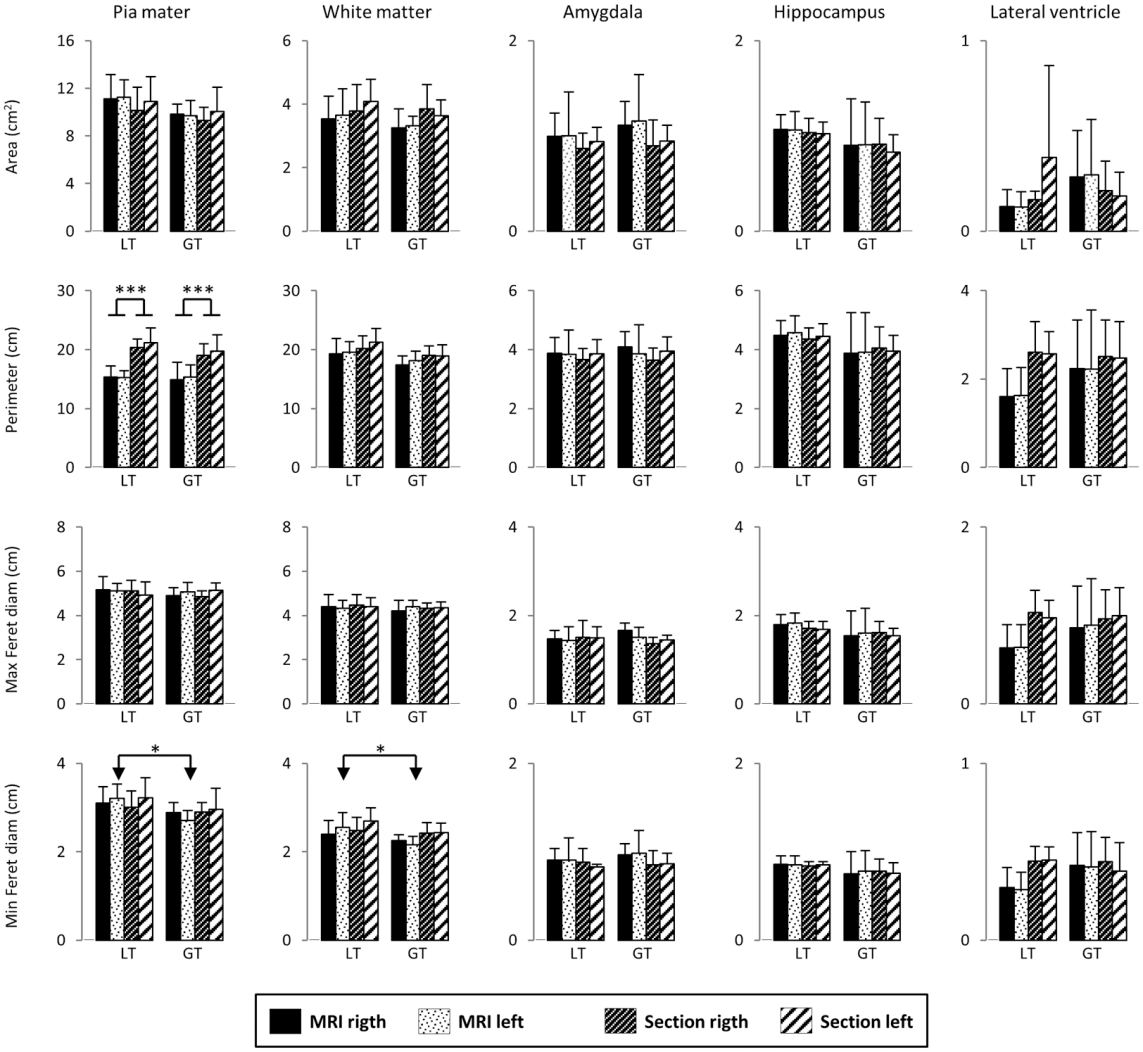
Plot of the size descriptors for the piamater, white matter, amygdala, hippocampus and temporal horn of the lateral ventricle sorted by groups of age (LT, lower than 65 years, and GT, greater than 65 years, see text) in both MRI and histological studies, as well as for both right and left hemispheres. Data represent mean and standard deviation.

**Fig 4:**
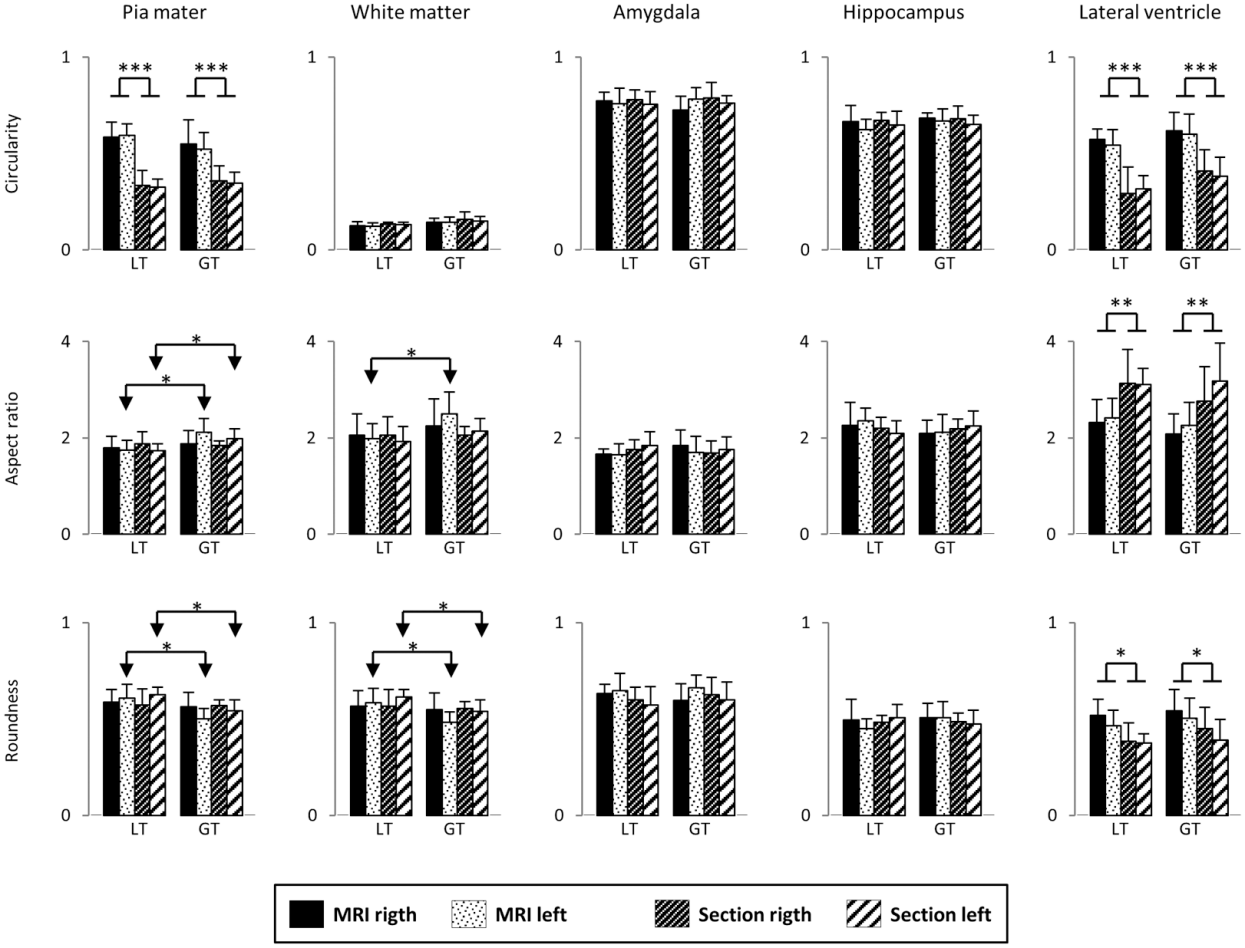
Size descriptors plot of the piamater, white matter, amygdala, hippocampus and temporal horn of the lateral ventricle by age group (LT and GT), in both MRI and histological studies, and for both right and left hemispheres. Data are mean and standard deviation.

The morphometric results obtained for piamater, white matter boundary and profiles of the amygdala, HF and lateral ventricle are shown in Figs 3 and 4 (size and shape descriptors, respectively). None of the descriptors showed statistical differences between hemispheres for both MRI and histological studies (mean and SD values are shown in Figs 3 and 4).

The average area per section (profile of the temporal lobe, piamater) was about 11 cm^2^ per hemisphere irrespectively of the method employed, MRI or histology (Fig 3). MRI perimeter values showed a significant decrease in both hemispheres relative to the histological analysis; this decrease appeared in both LT and GT groups. Minimal Feret’s diameters resulted in a small differences (p<0.05) between MRI and histology in the left hemisphere for both LT and GT groups. The piamater appeared more elliptical and irregular in histological sections than in MRI; besides, the circularity factor was significantly lower in histological sections than MRI values (Fig 4), and the aspect ratio (FFEll) and roundness changed significantly between age groups (p<0.05).

The white matter area presented values of about 4 cm^2^, while the perimeter was 20 cm (Fig 3). No significant differences were found between MRI and histology values. A small difference in the white matter (p<0.05) in minimal Feret’s diameters between LT and GT groups was found. Circularity values in the white matter were clearly lower than those of the piamater in both methods of study, age groups and hemispheres (Fig 4).

The average data of sectional area for the amygdala, HF and lateral ventricle were 0.85 cm^2^, 1 cm^2^, and 0.12 cm^2^, respectively (Fig 3). Descriptors of size and shape of amygdala and HF in the right and left hemispheres were not statistically significant; likewise, no statistical differences in both MRI and histological sections were observed. In contrast, the lateral ventricle showed the largest variability among all the regions analyzed, and presented shape descriptors with significant difference between MRI and histological sections in both LT and GT groups (Fig 4). The circularity index showed a decrease in histology relative to MRI.

Sex had no effect neither on size or shape descriptors in any of the MTL structures studied. Likewise, sex had no effect either in MRI or histological sections.

Table 2 shows the cortical thickness of the entorhinal and temporal cortices. No effect of age or sex on cortical thickness or hemispheres was observed. Moreover, the MRI study of the temporal lobe cortices showed a thickness smaller than in the histological study (12% of decrease, not statistically significant). The cortical thickness of the EC was 18% greater than temporal cortex although, it did not reach statistical differences.

**Table 2:** Entorhinal and temporal lobe cortices thickness in both, hemispheres and series.

			Thickness (mm)	
			Hemisphere	
			Right	Left
**Entorhinal cortex**	MRI	LT	2.850 ± 0.598	3.185 ± 0.635
	study	GT	3.307 ± 0.955	3.029 ± 0.877
	Histological	LT	3.017 ± 0.796	3.565 ± 0.421
	study	GT	3.666 ± 0.551	3.531 ± 0.425
**Temporal cortex**	MRI	LT	2.450 ± 0.491	2.475 ± 0.566
	study	GT	2.549 ± 0.654	2.460 ± 0.489
	Histological	LT	3.081 ± 0.347	2.846 ± 0.192
	study	GT	2.851 ± 0.290	2.820 ± 0.180
**Entorhinal cortex**	MRI	Men	2.903 ± 0.776	3.351 ± 0.697
	study	Women	3.204 ± 0.847	2.933 ± 0.761
	Histological	Men	3.276 ± 0.693	3.625 ± 0.358
	study	Women	3.388 ± 0.815	3.494 ± 0.453
**Temporal cortex**	MRI	Men	2.805 ± 0.505	2.658 ± 0.565
	study	Women	2.281 ± 0.508	2.331 ± 0.449
	Histological	Men	3.064 ± 0.360	2.896 ± 0.210
	study	Women	2.896 ± 0.310	2.788 ± 0.152

Age’s group: LT (lower than), less or equal to 65 years; GT (greater than), 65 years. Sex group: Men and Women.

Sex, age or hemisphere presented no statistical differences in the stereological estimates of the volume and surface area of the white and grey matter of temporal cortex, amygdala, HF and temporal horn of the lateral ventricle as shown in Tables 3 and 4 (age groups and sex, respectively). On average, MRI volume was 31 cm^3^ for the grey matter, and 25 cm^3^ for white matter. Histological measurements resulted in higher volume in grey matter (40 cm^3^) whereas white matter reached only 28 cm^3^. The variability of grey and white matter among cases, as well as temporal lobe volume was higher in histological sections than in the MRI study (on average, the coefficient of variation was 25.9% and 17.8%, respectively). The volume of amygdala, HF and lateral ventricle were, on average, 1.26 cm^3^, 3.28 cm^3^, and 1.02 cm^3^, respectively. The histological series had greater values in both hippocampi and lateral ventricle relative to MRI (with differences in the hippocampus that were statistically significant in some cases, see Table 3). The largest variability was found in the lateral ventricle. The surface area was also higher in histological measurements compared to MRI especially in the most irregular structures; moreover, these differences were more evident in the right hemisphere.

**Table 3:** Volume and surface area estimations for all structures analyzed in the temporal lobe of both hemispheres and series for age’s group (LT and GT cases).

			Volume (cm^3^)		Surface area (cm^2^)	
			Hemisphere		Hemisphere	
			Right	Left	Right	Left
**Temporal lobe**	MRI	LT	63.29 ± 6.00	62.08 ± 11.49	---	---
	study	GT	60.78 ± 4.35	63.62 ± 9.05	---	---
	Histological	LT	73.47 ± 18.66	85.85 ± 25.09	---	---
	study	GT	65.83 ± 10.28	61.57 ± 15.41	---	---
**White matter**	MRI	LT	28.08 ± 6.22	26.08 ± 6.59	115.2 ± 31.1	104.0 ± 37.1
	study	GT	23.27 ± 2.75	23.42 ± 3.93	106.3 ± 12.9	109.4 ± 16.9
	Histological	LT	28.10 ± 10.70	33.44 ± 13.25	129.1 ± 34.9	147.8 ± 37.3
	study	GT	23.33 ± 5.72	21.89 ± 5.61	126.0 ± 18.1*	117.1 ± 33.8
**Grey matter**	MRI	LT	30.06 ± 4.47	32.22 ± 10.71	163.0 ± 83.0	145.1 ± 60.2
	study	GT	32.72 ± 2.06	35.50 ± 4.77	144.0 ± 33.3	146.3 ± 37.9
	Histological	LT	39.30 ± 8.28	45.01 ± 11.32	224.2 ± 115.1*	262.0 ± 129.0*
	study	GT	36.05 ± 5.43	33.56 ± 8.76	205.4 ± 87.9	182.8 ± 83.1
**Amygdala**	MRI	LT	1.37 ± 0.33	0.97 ± 0.49	13.02 ± 3.48	4.53 ± 1.01
	study	GT	1.43 ± 0.34	1.22 ± 0.53	7.88 ± 1.91	4.78 ± 1.25
	Histological	LT	1.18 ± 0.50	1.37 ± 0.40	4.26 ± 2.35	5.52 ± 2.50
	study	GT	1.24 ± 0.06	1.30 ± 0.20	5.45 ± 0.20	5.50 ± 0.86
**Hippocampus**	MRI	LT	3.15 ± 1.03	2.30 ± 0.79	13.02 ± 3.48	10.37 ± 2.25
	study	GT	2.72 ± 0.74	2.92 ± 1.61	7.88 ± 1.91	9.91 ± 3.46
	Histological	LT	3.62 ± 0.62	4.54 ± 0.90**	15.54 ± 5.51	16.58 ± 8.39
	study	GT	3.68 ± 0.57**	3.33 ± 1.44	16.19 ± 2.49***	13.52 ± 6.02
**Lateral ventricle**	MRI	LT	0.62 ± 0.44	0.52 ± 0.26	7.12 ± 3.52	5.76 ± 1.50
	study	GT	0.63 ± 0.25	0.57 ± 0.29	5.52 ± 2.35	5.96 ± 1.86
	Histological	LT	1.28 ± 0.75	1.49 ± 1.12	11.17 ± 5.29	10.20 ± 5.19
	study	GT	1.53 ± 1.21	1.50 ± 1.22	10.93 ± 6.39	11.19 ± 7.68

Data are significantly different between MRI and histological studies;

*, at level of <0.05;

**, at level of <0.01;

***, at level of <0.001.

Age’s group: LT (lower than), less or equal to 65 years; GT (greater than), 65 years.

**Table 4:** Volume and surface area estimations for all structures analyzed in the temporal lobe of both hemispheres and series for sex group (Men and Women cases).

			Volume (cm^3^)		Surface area (cm^2^)	
			Hemisphere		Hemisphere	
			Right	Left	Right	Left
**Temporal lobe**	MRI	Men	61.59 ± 6.58	64.70 ± 12.25	---	---
	study	Women	62.36 ± 4.45	61.53 ± 8.63	---	---
	Histological	Men	61.67 ± 14.58	81.22 ± 20.96	---	---
	study	Women	75.35 ± 13.21	68.34 ± 25.37	---	---
**White matter**	MRI	Men	25.32 ± 5.55	25.17 ± 4.81	113.8 ± 8.9	117.8 ± 26.8
	study	Women	25.93 ± 5.42	24.45 ± 6.08	108.6 ± 30.3	98.8 ± 27.3
	Histological	Men	21.53 ± 9.04	29.46 ± 10.59	123.9 ± 30.0	155.3 ± 36.5
	study	Women	28.70 ± 7.38	26.39 ± 12.65	130.2 ± 26.0	116.2 ± 30.9
**Grey matter**	MRI	Men	31.42 ± 5.37	35.84 ± 10.59	165.5 ± 64.1	152.5 ± 25.3
	study	Women	31.37 ± 2.12	32.44 ± 6.29	145.0 ± 62.5	140.9 ± 61.1
	Histological	Men	34.28 ± 6.53	44.25 ± 9.85	214.9 ± 106.8	270.0 ± 133.9
	study	Women	40.10 ± 6.49	35.74 ± 11.68	214.7 ± 100.2	188.4 ± 87.0
**Amygdala**	MRI	Men	1.30 ± 0.42	0.90 ± 0.51	4.44 ± 1.46	4.83 ± 0.90
	study	Women	1.47 ± 0.24	1.23 ± 0.49	5.26 ± 0.90	4.54 ± 1.27
	Histological	Men	1.05 ± 0.44	1.32 ± 0.39	4.66 ± 2.00	5.99 ± 1.84
	study	Women	1.32 ± 0.22	1.34 ± 0.26	4.99 ± 1.63	5.17 ± 1.80
**Hippocampus**	MRI	Men	2.71 ± 0.64	2.12 ± 0.60	9.26 ± 3.03	9.43 ± 2.87
	study	Women	3.10 ± 1.05	2.96 ± 1.51	11.30 ± 4.27	10.64 ± 2.84
	Histological	Men	3.67 ± 0.54	4.61 ± 1.00	16.58 ± 2.02	19.48 ± 6.34
	study	Women	3.64 ± 0.63	3.45 ± 1.35	15.36 ± 5.22	11.88 ± 6.22
**Lateral ventricle**	MRI	Men	0.83 ± 0.33	0.68 ± 0.19	8.14 ± 3.38	6.42 ± 1.22
	study	Women	0.49 ± 0.29	0.45 ± 0.29	5.02 ± 1.96	5.46 ± 1.83
	Histological	Men	1.13 ± 0.54	1.59 ± 1.04	11.21 ± 3.65	12.77 ± 5.00
	study	Women	1.59 ± 1.20	1.41 ± 1.24	10.94 ± 6.97	9.21 ± 7.01

**Fig 5:**
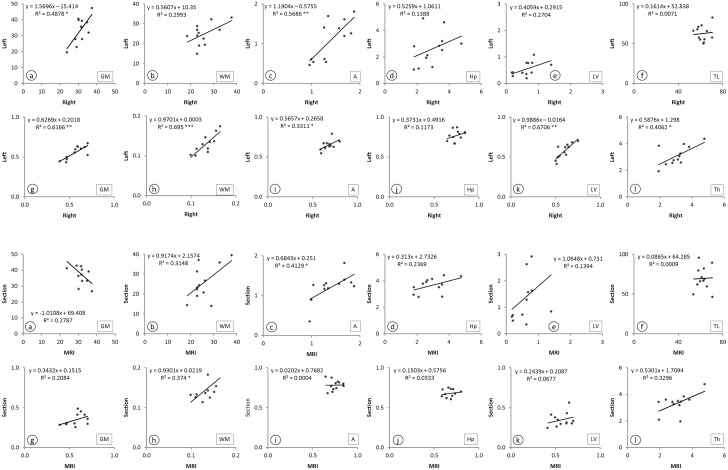
Plot of the volume (a-f), circularity form factor (g-k), and thickness (l) for the twelve studied cases; regression equation, correlation significance, and regression lines that fits the data are displayed. Hemispheric correlation (right and left) are shown in top, for MRI study. Correlation between MRI and histological measurements are shown at bottom, for right hemisphere. GM: Grey matter; WM: White matter; A: Amygdala; Hp: Hippocampus; LV: Lateral ventricle; TL: Temporal lobe; Th: Thickness (entorhinal cortex).

Overall, the study of correlation between parameters demonstrates correlation between quantitative estimators, although with high variability due to the high number of parameters in both groups. The results indicate greater association between hemispheres in both, size and form estimators than the MRI and histology methods of study (Fig 5). However, for each parameter type (namely volume and surface area) the multivariate analysis revealed a significant difference only for the factor method employed (no significant differences were detected in sex and age factors). Separate ANOVA analyses indicated that differences in the method affected mainly the hippocampus (p<0.01), and to a lesser degree the lateral ventricle (p<0.05), whereas in grey matter, white matter, and amygdala no significant difference was detected between the two methods. A separate univariate ANOVA was performed on the cortical thickness with no significant effects of any of the three factors considered.

## Discussion

The importance of quantitative analysis of the MTL has been demonstrated in a large quantity of studies of the human temporal lobe in normal and pathological conditions [9, 22–24]. However, studies are generally restricted to either MRI or neuroanatomical evaluation based on histological sections, but seldom combined [18]. We deemed interesting the study of the comparison of quantitative data obtained from MRI with data obtained by histological analysis of the MTL in the same subjects. Nowadays, non-invasive MRI methods are highly used in clinical practice to interpret morphologic changes in the brain, capable to detect neurodegenerative disorders at an early stage. In addition, our results show that in control cases, the quantitative data of the MTL are similar between hemispheres, sex, and age groups. Earlier studies [6] show a decrease in the calculated area of two-dimensional, unfolded maps in subjects over 80 years old, but in our series the number of cases of this age is too small to detect differences. It would be interesting to analyze more complete series of subjects older and younger than 80 years. Likewise, it would be interesting to establish hemispheric differences when neurodegenerative processes are diagnosed [4, 25].

A principal finding of this study is the high correlation between MRI and histological sections in quantitative measurements of size and shape descriptors. While it is possible that variability could change according to the number of images used for quantification (probably higher when fewer sections are employed) the values of area, perimeter and Feret’s diameters were roughly similar between both MRI and histology; only the perimeter of the white matter and, in particular of the piamater showed a statistically significant increase in histological sections relative to MRI. This fact could be explained by a more clear outlines of histological sections that would allow a more accurate tracing of brain sulci. MRI, as it has been applied here, showed partial volume effect and did not give clear definition of the depth of some sulci, although no significant changes in area measurements were obtained despite an increase in the perimeter. Likewise, the lateral ventricle had more clear limits on histological material than on MRI sections, what resulted in an increased perimeter, especially in LT group. Both cases (piamater and lateral ventricle) had the value of the circularity index significantly reduced, thus indicating higher irregularity.

The importance of simple and easy to perform measurements of the cortical thickness to differentiate controls from neurodegenerative disorders patients has been reported in many studies [26–29]. Our results deal exclusively with control cases, in which EC thickness values are in the range of other reports [26, 30]. However, the temporal cortex thickness is a bit smaller than the EC. We found a notable, but not significant decrease of the cortical thickness measured in histological sections compared to MRI studies; this fact could be explained, in part, by the lack of contrast of the cortex in MRI images, as the close contact between adjacent banks of the sulci results in a fused mass of tissue, where precise limits cannot be seen (partial volume effect), thereby the outlines are better identified on histology than in 1.5T MRI. Some studies found an increase of the cortical thickness of the MTL, whose values were slightly greater in perirhinal, parahippocampal and fusiform gyrus cortices relative to EC [26], although several authors report a thickness decrease in regions outside the EC [27, 30, 31].

Much larger left hemisphere regional brain volume has been described [32], thus indicating a clear asymmetry, including temporal regions. Our results show the temporal lobe volume estimation similar in both hemispheres, with values in accordance to other studies using 1.5T MR images [33, 34]. Moreover we found that sex and age groups did not present a significant influence on volume estimations for any of the temporal lobe regions analyzed. Sex differences were reported in several studies after analysis of volume and size distribution of neocortical neurons, as well as neuron numbers in brain regions [35–38]. Different reports exist dealing with sex influence, mainly related with brain region studied, normalization of the brain size, and methodology, as Keeley *et al*. report [36]. Our study shows data variability when different compartments were measured that, in addition to the scarce number of cases studied, resulted in no sex differences, being in accordance with the more exhaustive study of Insausti *et al*. [6] on the no influence of sex when analyzing temporal cortices after normalization of volumetric data.

Our study shows an increase of roughly 13% of the temporal lobe volume estimation using histological material relative to MRI, which was more evident in grey than in white matter. Surface area had more clear-cut, statistically significant value differences in histological analysis relative to MRI (16% and 30% of increase in white and grey matter, respectively); these findings could be due to a more accurate identification of boundaries, whereas MRI images, at least with 1.5T scanners, present poor contrast of boundaries in temporal lobe structures. Surface area estimation on post-mortem brains gave similar values [39]. Smaller values of surface area in MRI relative to histology are more pronounced in the outer, pial surface, and our results are confirmatory [39].

The volumetric data of hippocampus, amygdala, and lateral ventricle show almost no variability between hemispheres. Only the hippocampus presented in some cases statistically significant differences between MRI studies and histology. While hemispheric asymmetry, mainly using MRI has been reported extensively, fewer studies exist in which the effect of sex is included [14, 40–45]. Our results indicate that no effect on morphometric and volumetric data are due to sex.

The volume of hippocampus is around 3–4 cm^3^ (range 2 to 8.5 cm^3^) in most studies [46–49]. The amygdala varies between 1–2 cm^3^ (range 0.6–4 cm^3^). Only a few studies report the volume of the temporal horn of the lateral ventricle (values around 0.3 cm^3^) [50]. Finally, the resulting surface area values showed that histological studies provide higher values relative to MRI, especially in the hippocampus and lateral ventricle, where higher inter-individual variability was found. Consequently, it results in statistically significant differences, likely due to the number of intercepts between structures and test lines. The surface area estimation requires isotropy to obtain unbiased data; however, although in our study only coronal sections were used (what produces a non-random orientation, and therefore non-unbiased data), we think, such as Chareyron *et al*. [51] and Jabés *et al*. [52], that since the analysis of the brain structures under study was made in the same way in both MRI, and histology, data collected in the study would allow the assessment of surface area measurements.

The correlation and the multivariate studies revealed that an association exists between quantitative data of MRI and histological material, with no influence of sex and age groups. However, this association was not extremely significant, perhaps due to the small number of human cases analyzed and the individual variability. The MANOVA analysis reveals a significant difference between both methods for volume and surface area estimations in the hippocampus and lateral ventricle that are also in concordance with the fact that MTL structures with irregular morphology tend to be influenced in a larger degree than those with less variability.

We found that the use of test points for volume estimation is more efficient (i.e., 1/(coefficient of variation•time)) than the use of regions of interest, usually obtained by manual tracing and the subsequent product of surface by number of sections; however, more advanced methods such as statistical parametric mapping (SPM) and FreeSurfer software systems for automatic tracing improve the delineation of regions of interest and, thus, increase the efficiency due to reduction of time. We tested this methodology in other studies with promising results [53, 54], and Lehman *et al*. [22] demonstrate the accuracy of FreeSurfer software to identify atrophy and distinguish temporal lobe structures between groups such as AD, semantic dementia, and control subjects [22]. However, in spite of these results with this software, we used simple count of test points or linear intersections due to the proven efficacy in stereology.

In conclusion, we show that a simple, easy to implement, unbiased method for volumetric assessment of MRI images, can be applied to clinical studies, in particular to the MTL and structures therein, with an overall accuracy of a histopathological estimation of the same volumes as morphometric parameters.
